# Nanohydroxyapatite-Mediated Imatinib Delivery for Specific Anticancer Applications

**DOI:** 10.3390/molecules25204602

**Published:** 2020-10-09

**Authors:** Paulina Sobierajska, Anna Serwotka-Suszczak, Damian Szymanski, Krzysztof Marycz, Rafal J. Wiglusz

**Affiliations:** 1 Institute of Low Temperature and Structure Research, Polish Academy of Sciences, Okolna 2, 50-422 Wroclaw, Poland; d.szymanski@intibs.pl; 2 Department of Experimental Biology, Wroclaw University of Environmental and Life Sciences, Norwida 27B Street, A7 Building, 50-375 Wroclaw, Poland; anna.serwotka-suszczak@upwr.edu.pl (A.S.-S.); krzysztofmarycz@interia.pl (K.M.); 3 International Institute of Translational Medicine, Malin, Jesionowa 11, 55-114 Wisznia Mała, Poland; 4 Faculty of Veterinary Medicine, Equine Clinic-Equine Surgery, Justus-Liebig-University, 35392 Giessen, Germany

**Keywords:** nanohydroxyapatite, imatinib, anticancer targeted therapies, cytotoxicity

## Abstract

In the present study, a nanoapatite-mediated delivery system for imatinib has been proposed. Nanohydroxyapatite (nHAp) was obtained by co-precipitation method, and its physicochemical properties in combination with imatinib (IM) were studied by means of XRPD (X-ray Powder Diffraction), SEM-EDS (Scanning Electron Microscopy-Energy Dispersive X-ray Spectroscopy), FT-IR (Fourier-Transform Infrared Spectroscopy), absorption spectroscopy as well as DLS (Dynamic Light Scattering) techniques. The obtained hydroxyapatite was defined as nanosized rod-shaped particles with high crystallinity. The amorphous imatinib was obtained by conversion of its crystalline form. The beneficial effects of amorphous pharmaceutical agents have been manifested in the higher dissolution rate in body fluids improving their bioavailability. Imatinib-to-hydroxyapatite interactions on the surface were confirmed by SEM images as well as absorption and FT-IR spectroscopy. The cytotoxicity of the system was tested on NI-1, L929, and D17 cell lines. The effectiveness of imatinib was not affected by nHAp modification. The calculated IC_50_ values for drug-modified nHAp were similar to those for the drug itself. However, higher cytotoxicity was observed at higher concentrations of imatinib, in comparison with the drug alone.

## 1. Introduction

Receptor tyrosine kinases (RTKs) are a large group of enzymes involved in a critical number of processes necessary for the proper functioning of both cells and organisms. Since RTKs coordinate a wide variety of cellular functions, such as cell proliferation and differentiation, their activity must be regulated to prevent severe abnormalities in cellular functioning. Activated forms of the RTKs may be caused by an increase in the proliferation and growth of cancer cells, induce antiapoptotic effects, and promote angiogenesis and metastasis. Somatic mutations resulting in the constitutive activation of protein kinases are a common mechanism for cancer formation, often independent of ligand binding [1].

For the above reasons, receptor tyrosine kinases are key for anticancer targeted therapies. Many tyrosine kinase inhibitors (TKIs) have been designed so far, and many of them have clinical applications. The United States Food and Drug Administration (FDA) has approved several drugs that target cancer caused by the activated RTKs; most of them are binding the extracellular or catalytic domain and inhibit ligand binding as well as receptor oligomerization. Imatinib was first developed against chronic myelogenous leukemia (CML) and followed by gefitinib and erlotinib targeted at the EGF receptor. Sunitinib is an inhibitor of FGF, PDGF, and VEGF receptors that is also based on early TKI-targeted research on VEGF receptors [2,3,4,5].

Even though the TKIs are highly effective against some kinase receptor-dependent cancers, their utility is seriously compromised by some of their serious disadvantages. Their therapeutic efficacy may be restricted by their limited oral bioavailability associated with poor aqueous solubility, permeability, and extensive plasma protein binding capacity. The treatment may be accompanied by a series of side effects, including rash, erythema, diarrhea, gastrointestinal perforations, ocular lesions, and hematological disorders. Despite the significant initial response, the long-term effectiveness of TKI therapy, it may be limited by the development of acquired resistance in most patients. To overcome this obstacle, new generations of the TKIs have been developed by modification of the structure of the primary drugs [1,6,7,8,9,10,11,12].

To improve the TKIs properties and overcome their limitations, the possibilities of drug conjugates, hybrid drugs, or nanoparticle-based drug delivery systems are being investigated. Various delivery systems at the nanoscale seem to not only help solve basic imperfections but also increase efficacy through selective accumulation in tumor tissues. Nanoparticles can penetrate leaking blood vessels and accumulate in tumor tissues in the process of so-called “passive” transport [12,13,14,15].

Among many different types of nanoparticles, hydroxyapatite (herein nHAp, Ca_10_(PO_4_)_6_(OH)_2_) has been attracting great interest due to its outstanding biocompatibility, bioactivity as well as the lack of inflammatory and immunological responses [16]. Synthetic hydroxyapatite has a similar structure and grain size with natural hydroxyapatite; therefore, nHAp has been extensively developed as bioceramics, in the case of the reconstructs damaged parts of hard tissues of the skeletal system [17]. nHAp has been mainly used as bone graft material or scaffold and as an implant coating layer. Applications include maxillofacial reconstruction, treatment of periodontal disease as well as bone repair after tumor surgery due to its ability to promote bone ingrowth [18]. Our previous work showed that synthetic nanocrystalline hydroxyapatite has proper biocompatibility, even after structural modification by ionic substitutions [19]. The biological activity of nanoapatite (as well as other nanoparticles) results from its surface chemistry associated with high surface area to volume ratio. Thus, the surface functionalization of nHAp with biologically active molecules makes it suitable as a drug carrier of chemotherapeutics and antibiotics [20,21,22].

Besides the biomolecular approach, physicochemical properties such as size, shape, and surface charge play an important role in biodistribution, cellular internalization, and intracellular trafficking of the nanoparticles [23,24,25]. Many studies indicate that spherical nanoparticles exhibit appreciable cellular uptake [26,27]. Moreover, the other research has shown that rod-like HAp nanoparticles could be more easily uptaken by the cells [25], and nanoparticles with positive charge have a greater affinity towards cellular membranes [26]. It seems to be a very important item, especially for usually negatively charged cancer cells [28]. The strategy of functionalization the surface of the nanoparticles may enhance their internalization with tumor cells making the therapeutic delivery system more precise and effective. The size of the nanoparticles also plays a pivotal role in their adhesion and interaction with the cells. However, there is no definitive answer as to which particle size will be most favorable. It depends on specific mechanisms of the passage of particles through the physiological barriers, as well as the type of cells treated [25,29].

Loading drugs onto nanoparticle surfaces is a relatively new concept of the targeted systems in the case of chemotherapy as well as other disease treatments. In this study, we explored the possibility of combining nHAp with well-described TKI, imatinib, as well as the effect on drug effectiveness. Imatinib has been approved by the FDA since 2001 and can be used to treat several types of RTKs-associated cancers, including ABL (Tyrosine-protein kinase ABL1), BCR-ABL (fusion gene of breakpoint cluster region and Tyrosine-protein kinase ABL1 genes), PDGFRA (platelet-derived growth factor receptor A), and c-Kit tyrosine kinases. The mechanism of action of imatinib is well known and based on the inactivation of protein kinase overexpression by competitive binding close to the ATP active site [30,31]. In this paper, a new system of synthetic nanocrystalline hydroxyapatite (nHAp) and its imatinib-modified version is proposed for biomedical applications in the case of cancer therapy. For this purpose, the influence of these compounds on the viability of canine mastocytoma NI-1 cell line, which is positive for the presence of a mutation in the c-Kit tyrosine kinase, was investigated. The effect of imatinib on this cell line has already been evaluated [32]. Thus, this research demonstrates the extremely important role of the structural and morphological parameters of nHAp for specific nanoparticles-based anticancer applications.

## 2. Results and Discussion

### 2.1. Characterisation of nHAp, Imatinib and nHAp/Imatinib

Amorphous substances, compared to their crystalline forms, possess a more irregular shape as well as higher internal energy and specific volumes, improving the dissolution and bioavailability of these compounds [33]. It means that the same drug could be used at a lower dose leading to a reduced risk of local side effects caused by unabsorbed materials and has a cost-saving effect. In a physicochemical point of view, amorphous substances (with no long-range order of molecular packing or well-defined molecular conformation in contrast to crystalline solid) are generally less stable than corresponding crystals. However, if there are those that exhibit greater stability, they could constitute a potential novel drug product. One of them is amorphous imatinib (IM-4-[(4-Methyl-1-piperazinyl)methyl]-*N*-[4-methyl-3-[[4-(3-pyridinyl)-2-pyrimidinyl]amino] phenyl]benzamide methanesulfonate) which has confirmed long-term stability [33]. imatinib (IM) is tyrosine-kinase inhibitor use in certain types of cancer treatments, e.g., myeloid leukemia [34], gastrointestinal stromal tumors [35], and also in the other malignancies involving the expression of tyrosine kinase [30]. It is one of the first compounds used effectively in molecularly targeted therapies. The imatinib occurs in several polymorphic states like the most common α- and β-forms [36]. Some studies have shown that the α-IM (described as metastable) is not useful for the pharmaceutical formulation preparations because of its high hygroscopic properties. Whereas, the β-form possesses a lower hygroscopic ability and higher flow properties, as well as higher thermal stability than the α-form [37]. Nevertheless, there are other IM phases that are still better for manufacturing, pharmaceutical preparation, and storage.

**Figure 1:**
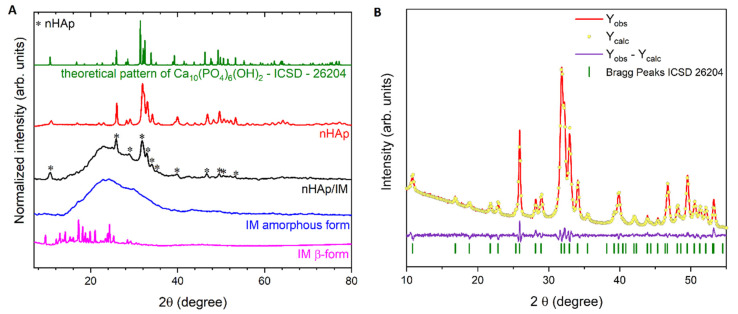
X-ray Powder Diffraction (XRPD) patterns (**A**) of the crystalline imatinib (IM) β-form (magenta line), amorphous IM (blue line), nHAp modified by imatinib (nHAp/IM, black line), and nHAp (red line) with the corresponding reference hydroxyapatite pattern (ICSD–26204). Rietveld analysis (**B**) of the obtained nanohydroxyapatite (nHAp).

The crystalline IM using in this study (supplied by Sigma Aldrich) represents the β-form that has been measured by the X-ray Powder Diffraction (XRPD) technique (see Figure 1A, magenta line). The observed diffraction peaks at 2θ values correspond very well to the literature [37]. The β-form has characteristic peaks located at 9.7°, 13.9°, 18.2°, 20.0°, 21.1°, and non-observed peaks at 4.9°, 10.4°, 18.6°, 24.8°, which are commonly assigned to the α-form [37,38]. Surprisingly, it was found that after preparation of the drug for biological studies, IM was transformed into an amorphous form. A broad halo with the absence of any sharp diffraction signals (correlate to reflexes observed for a crystalline form) could be seen in the XRD pattern (see Figure 1A, blue line), characteristic of amorphous material. The X-ray powder diffraction patterns of pure nanohydroxyapatite and nHAp/IM are represented in red and black lines in Figure 1A, respectively. As can be seen, the pure hexagonal phase corresponding to the standard reference (green line) of the Ca_10_(PO_4_)_6_(OH)_2_ (ICSD–180315) was observed as well as after surface modification with the drug. The observed peaks of the nHAp/IM pattern belonged to nHAp are located at 2θ equal to 10.7°, 25.9°, 28.9°, 31.9°, 32.9°, 34.0°, 35.2°, 39.8°, 46.8°, 49.6°, 50.7°, and 53.4°, while broad halos noticeable between 2θ 15°–40° originated from amorphous imatinib [39]. No other phases or impurities were observed.

The unit cell parameters of the obtained nHAp were calculated by means of the Rietveld refinement using an isotropic approach. The results are gathered in Table 1 and compared with the reference data. The small value of the refine factor (R_wp_) derived from the analysis identifies the success of the conducted refinement, and thus confirms the hexagonal structure of the studied hydroxyapatite. As can be seen in Figure 1B, there were clearly visible small differences in the intensity (Y_Obs_−Y_Calc_) between the observed XRPD pattern and the theoretical fit calculation.

**Figure 2:**
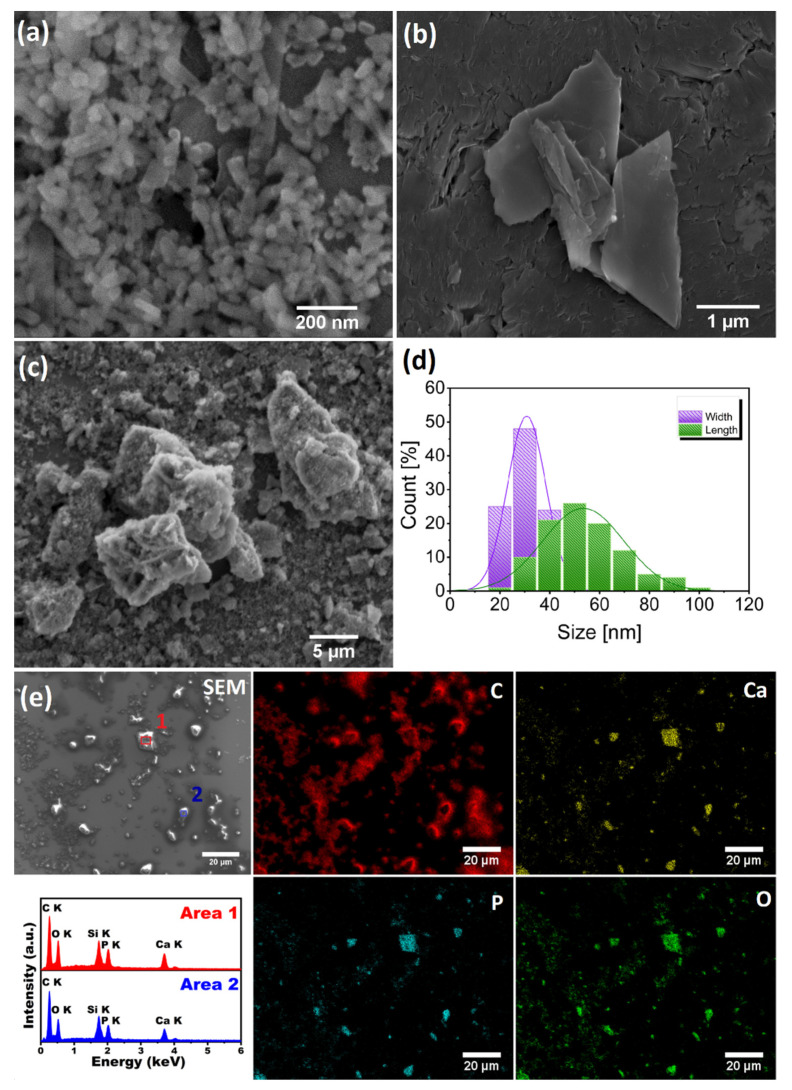
Representatives SEM images of the (**a**) nanohydroxyapatite (nHAp), (**b**) amorphous imatinib (IM) and (**c**) nHAp modified by imatinib (nHAp/IM), (**d**) histogram of the nHAp grain size distribution (length-wise and width-wise diameters), (**e**) EDS elemental mapping of the nHAp modified by imatinib together with EDS spectra for selected areas.

In order to determine the morphology of the nHAp and IM as well as nHAp/IM samples, scanning electron microscopy (SEM) was performed. Figure 2a–c) shows representative SEM images of the studied samples. As can be seen, the amorphous phase of imatinib resembled irregularly shaped flakes that are clearly different from the crystalline cubic-shaped β-form showing by Rohani et al. [37]. It is also clearly visible that nHAp particles have elongated rod-like shapes with mean particle sizes of 52 nm in length and 30 nm in width (see Figure 2d), which is close to the value obtained from Rietveld analysis (see Table 1). The particles exhibit a significant tendency to agglomeration. Moreover, no visual differences in the nHAp morphology have been detected after incubation with the drug compared with the unmodified material.

The next step was to apply SEM-EDS imaging to the identification of elements in both nHAp/IM components. As can be seen in Figure 2e, a scanning electron microscopy image (SEM) shows the sample morphology, while elemental maps of C, Ca, P, and O revealed the distribution of chemical elements in the sample. Carbon (C) atoms were used as a marker to confirm the presence of the drug in nHAp/IM because hydroxyapatite (nHAp) does not contain any C atoms in its structure (C). As was expected, the amount of C present in nHAp/IM comprised a maximum proportion of all the other elements. Oxygen (O) and calcium (Ca), as well as phosphorus (P) atoms, were detected, and these atoms were identified as components of the chemical structure of nHAp. The relative quantitative ratio of individual elements in nHAp/IM is presented in EDS spectra (see Figure 2e). The X-ray emission line with a maximum of 1.74 keV was also observed and assigned to the K_α_ line of Si (from silicon stub, see Materials and Methods section). The obtained contents of Ca and P in the studied composition were 11.9 at% (Area 1), 10.7 at% (Area 2), 7.1 at% (Area 1), and 6.6 at% (Area 2), respectively. The average Ca/P molar ratio has been estimated to be around 1.65, which was quite close to the theoretical value equal to 1.67. The data derived from the EDS analysis showed that surface interaction and incorporation of nHAp particles with drug molecules were taking place.

**Figure 3:**
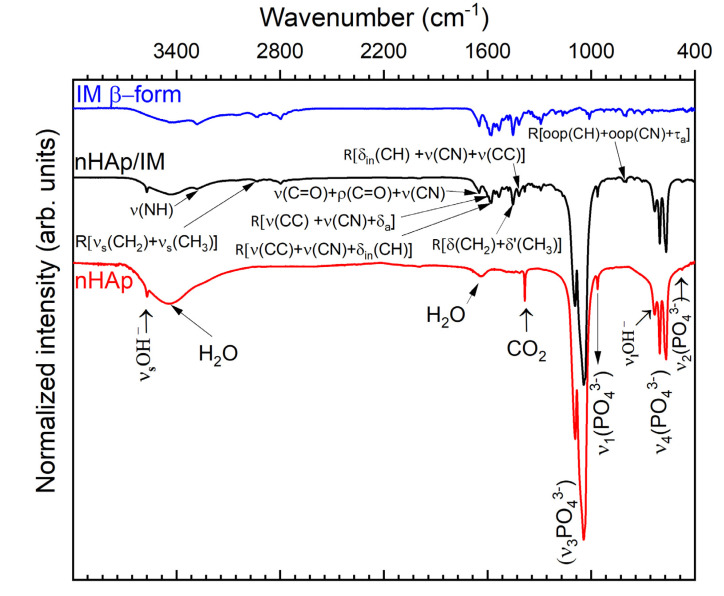
FT-IR spectra of imatinib (IM β-form, blue line), nanohydroxyapatite (nHAp, red line), and nHAp/IM (black line) with the indication of characteristic vibration bands for both compounds. Types of vibration: ν, stretching; δ, deformation (bending); oop, out-of-plane bending; ρ, rocking; τ, torsion.

To provide deeper analysis of the interaction between imatinib and Ca_10_(PO_4_)_6_(OH)_2_ nanoparticles, as well as its influence on composition, FT-IR measurements were conducted. The obtained results have been presented in Figure 3. The infrared spectrum of the nHAp/IM (black line) showed characteristic bands for both compounds [41,42]. The most intense infrared absorption bands for imatinib appeared at 2932 cm^−1^ (C-H stretching N-methylpiperazine ring vibrations), 1657 cm^−1^ (C=O stretching vibration mixed with C=O rocking vibration and C-N stretching vibration), 1574 cm^−1^ (C-C and C-N stretching pyridine and aminopyrimidine ring vibrations mixed with in-plane deformation of C-H), 1452 cm^−1^ (C-H symmetric and asymmetric deformations of the *N*-methylpiperazine ring), 1418 cm^−1^ (in-plane deformation of C-H mixed with stretching vibration of C-N of pyridine and aminopyrimidine rings, as well as C-C stretching methylbenzene ring vibration), and 804 cm^−1^ (out-of-plane bending mixed with asymmetric torsion of the methylbenzene ring). The band occurring at 1597 cm^−1^ was assigned to the C-C stretching mixed with C-N stretching and asymmetric deformation of the pyridine ring. While the broad band observed at 3275 cm^−1^ was associated with the N–H stretching vibration of open-chain amides in the imatinib solid state [43]. The IR spectrum of nHAp combined with imatinib consisted of typical active vibrational bands for nHAp structure [44] related to the phosphate PO_4_^3−^ groups located at 472.3 cm^−1^ (*v_2_*), 563.8 cm^−1^ and 603.9 cm^−1^(*v_4_*), 961.8 cm^−1^ (*v_1_*), as well as 1041.7 cm^−1^ and 1092.2 cm^−1^ (*v_3_*). The broad bands observed at 3445.8 cm^−1^ and 1634.7 cm^−1^ were associated with OH^−^ stretching vibration (*v(OH)*) from H_2_O adsorbed on the surface of nHAp. While the narrow band located at 1383.2 cm^−1^ was related to the vibrations of CO_3_^2−^ groups, indicating a trace amount of these anions. The bands at 633.2 cm^−1^ and 3574.0 cm^−1^ were assigned to librational (*ν_L_*) and hydroxyl stretch (*ν_S_*) modes of OH^–^ groups belonging to the nHAp crystal lattice, respectively. One can note, the FT-IR spectrum of nHAp/IM (see black line, Figure 3) was similar to both connected spectra of pure IM (blue line) and nHAp (red line). Therefore, this fact can be interpreted as a lack of significant chemical bonds between hydroxyapatite and modifier.

The FT-IR technique was also used for the definitive identification of imatinib polymorphs. The modes of α and β forms of imatinib did not show large deviations. However, certain differences have been observed between the wavenumber values mainly reflected for the N-H and C=O group in both forms [42]. The spectra of pure chemotherapeutic as well as nHAp/IM did not exhibit an additional vibrational band at 1446.0 cm^−1^ specific for the α‒form. The lack of detection related to moderate and weak bands in the 2706–2492 cm^−1^ spectral range could be characteristic of the imatinib β‒form [45]. Moreover, there have been no detected differences in spectra between the β-form (blue line, Figure 3) and amorphous (black line) form. Both spectra could be found due to broadened bands attributed to the amorphous IM, showed by Mucha et al. [33].

**Figure 4:**
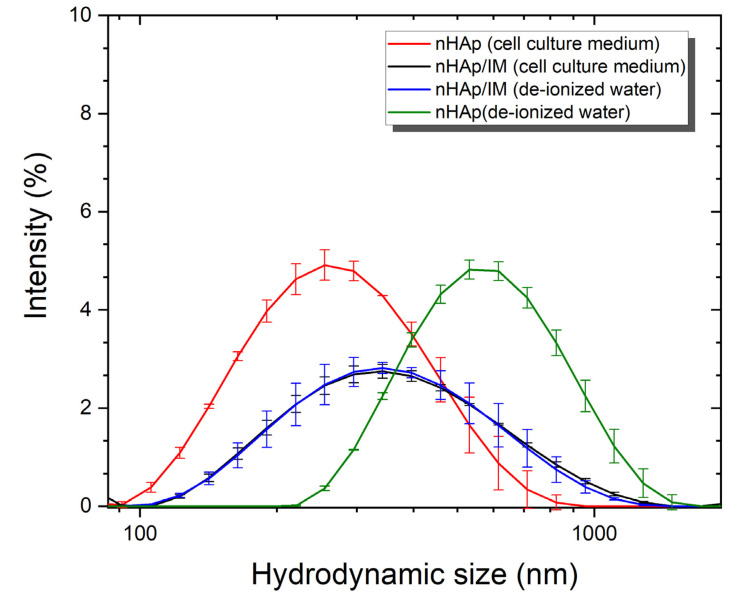
Hydrodynamic size of nanohydroxyapatite (nHAp) and nHAp modified by imatinib (nHAp/IM) monitored in de-ionized water and cell culture medium.

In fact, the susceptibility of cancer cells to nHAp/IM depends on the size, morphology, and surface charge of nanoparticles. Therefore, it is necessary to study the hydrodynamic particle size in a medium like body fluids. The performed observations (see Figure 4) using a size distribution profile revealed that cell culture medium significantly influenced the stabilization of the nHAp nanoparticles suspension. Surprisingly, after modification of nHAp with imatinib, a similar particle size profile was monitored in both (water and cell culture medium) suspensions. Thereby, the drug molecule clearly affected the degree of dispersion of the particles in the studied fluids. The typical hydrodynamic diameter of nHAp/IM was estimated to be around 345 nm and was associated with an affinity for particle agglomeration in the used media. Undoubtedly, the obtained nHAp showed a tendency for agglomeration, which was also confirmed by SEM images (see Figure 2). In the case of this study, the synthesis route resulted in relatively small nanoparticles. However, further research is required to work on the modification/functionalization of the nanoparticle surface for better dispersion in body fluids.

In cell biology, Zeta (ζ) potential is widely used to study the stability and surface charge associated with the capacity of cellular interactions with nanoparticles. In this study (see Table 2), the Zeta potential changes were observed and were particularly visible for systems differing in the type of the used medium. It should be noted that the cell culture medium strongly increased the value of the Zeta potential in comparison with de-ionized water. Negatively charged nanoparticles are repelled by the large, negatively charged cancer cell surface due to electrostatic interactions between them. One possibility for adjusting for the composition charge changes is to use a surface functionalization of the nanoparticles by small-molecule ligands, proteins, or antibodies, as well as polymer coatings such as polyethylene glycol (PEG) [46]. A reduced tendency to agglomerate can be achieved by grafting organic molecules containing ionic end-groups complexing surface calcium ions like carboxylates, phosphates, or phosphonates, and thus limiting crystal-crystal interactions due to steric hindrance. On the other hand, the electrostatic repulsion can be combined with the steric effect by selecting molecules that exhibit additionally other ionic end-groups, such as –NH_3_^+^. The promising colloidal apatite system was proposed by C. Drouet et al. [47], where apatite nanoparticles have been stabilized with a natural phospholipid moiety. Moreover, particle surface functionalization can make them attractive for therapeutic applications contributing to enhanced interactions with tumor cells and increase their uptake, thus making the drug delivery system more effective and targeted. The colloidal stability of the proposed system in biological environments is a challenging issue for future work.

Furthermore, one of the initial stages should be determined by the drug release profile. For this purpose, time-dependent imatinib release from the nHAp/IM was carried out in phosphate buffer (pH 7.4). The percentage of the released drug after 24 h is shown in Figure S1. As can be seen, the highest dose of IM (about 100%) was achieved after 45 min of nHAp/IM incubation in the physiological solution, and practically 88% of the drug was disconnected from nanoparticles during the first 5 min. It means that the imatinib release was almost instantaneous. The result is also expected in the context of the therapeutic effect, which would be better if more drug was released. The drug would be more accessible to surrounding tissues. However, it should be considered the drug availability, especially in the fluid exchange conditions through biological membranes. Our previous research indicates a more prolonged way of releasing chemotherapeutic when it passes through membranes [48]. It is also worth working on ligand attachment to nanoparticles to obtain a stronger connection with the drug and thereby getting a more constant release over a longer period. This approach could extend and consolidate the intended therapeutic effect. In this study, the drug-loading ratio was 2 wt%, and the loading efficiency was 6 wt% IM. Although the low loading capability of nHAp/IM limits its clinical application, the nHAp can be considered a promising platform for the delivery of targeted anticancer drugs, and it has been further investigated in cytotoxicity studies.

### 2.2. The Cytotoxicity Evaluation of the nHAp/IM

To analyze the influence of the combination of imatinib with nanohydroxyapatite on the effectiveness of the drug, the cytotoxicity of the compounds tested was examined for cells that are characterized by the presence of mutations in the tyrosine kinase receptor genes. The NI-1 line is a canine mastocytoma model for studying drug resistance that has confirmed mutations in the tyrosine kinase c-Kit. This line was well characterized and served as a model for imatinib toxicity testing [32]. The L929 murine fibroblast cell line was used as a control line for the experiment since it is a non-cancer cell line, with no known mutation in receptor tyrosine kinases. The D17 canine osteosarcoma cell line was chosen as a control cell line because there was no reference in the literature to the presence of c-Kit mutations nor the effect of imatinib on its viability. To evaluate the effect of experimental compounds, a range of eight different concentrations of imatinib alone and in combination with nano-hydroxyapatite (1 × 10^−11^ to 2.29 × 10^−5^ M for imatinib and 1 × 10^−10^ to 1 × 10^−10^ g/mL for nHAp) was assessed using TOX-8 assays, after 48 h of incubation.

**Figure 5:**
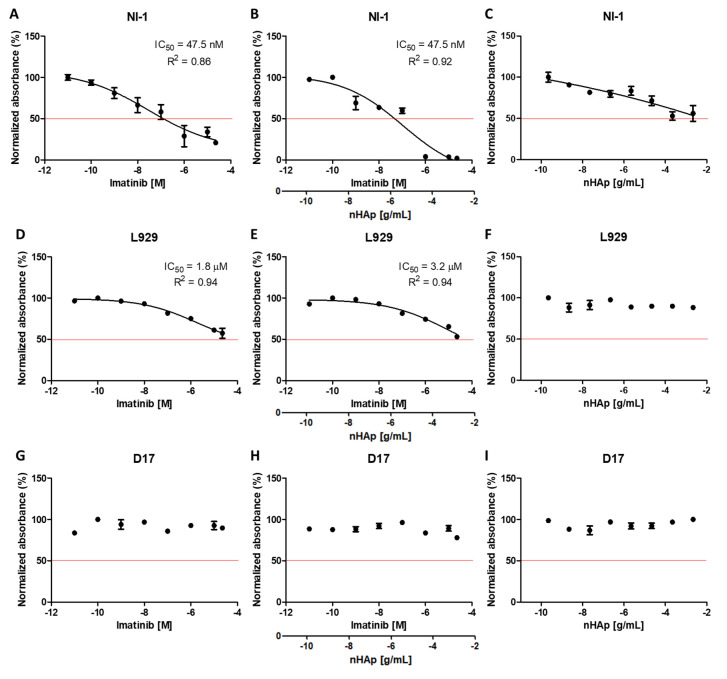
TOX-8 viability test results for NI-1 (**A**–**C**), L929 (**D**–**F**), and D17 (**G**–**I**) cells depending on the concentration of experimental compounds used: imatinib, nHAp modified with imatinib and nHAp alone. IC_50_ is indicated if possible. R^2^ stands for the coefficient of determination.

Figure 5 presents the results of the half-maximal inhibitory concentration (IC_50_), which was measured by the potency of a substance in inhibiting a specific biological or biochemical function—the metabolic activity of cells. The calculations were carried out using the GraphPad Prism 5.01 program. The calculated IC_50_ coefficient (together with the R^2^ determination coefficient) was marked in tests where its calculation was possible as a concentration-dependent effect on cell viability.

Graphs obtained for samples with nanohydroxyapatite alone suggest that nHAp alone had no toxic effect on lines L929 (Figure 5F) and D17 (Figure 5I), which is in line with earlier reports about its biocompatibility [48,49,50]. In the case of the NI-1 line, a decrease in viability was observed; however, even at the highest concentrations, cell viability did not fall below 50%.

**Figure 6:**
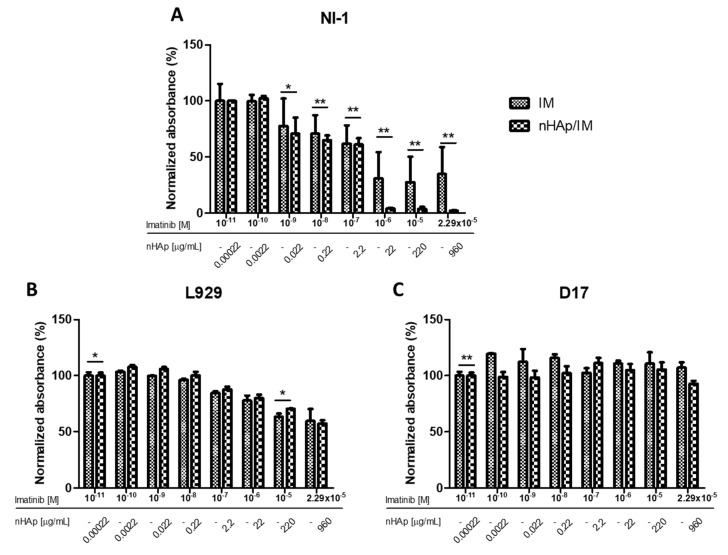
Comparison of results of cytotoxicity analysis of imatinib and modified nHAp on NI-1 (**A**), L929 (**B**), and D17 (**C**) cells. Results are expressed as mean ± SD, * *p* < 0.05, ** *p* < 0.01.

As has been expected, imatinib alone caused a significant decrease in cell viability of NI-1 (Figure 5A). The calculated IC_50_ was 47.5 nM, which was only slightly different from those presented by others (0.125–0.25 µM) [32]. For the same cell line treated with imatinib-modified nHAp (Figure 5B) the IC_50_ coefficient was identical (47.5 nM). However, it is worth noting that at higher concentrations, the decrease in viability in this test was much more observable than in the case of the drug alone, as has been shown in Figure 6A.

In the case of mouse fibroblasts from the L929 cell line, there was a tendency for imatinib (Figure 5D), as well as imatinib-modified nHAp (Figure 5E) to cause cell death at higher concentrations. The calculated IC_50S_ were 1.8 μM and 3.2 μM, respectively. Whereas the nano-hydroxyapatite applied alone seemed to be non-toxic (Figure 5F). The results were even more surprising because the line was non-cancerous and not characterized by the presence of known mutations in tyrosine kinase receptor genes. No difference between the effects of imatinib alone and in combination with nHAp on these cells was seen (Figure 6B).

The results obtained for the D17 control line suggest that all three treatments: drug alone (Figure 5G), imatinib-modified nHAp (Figure 5H) and nHAp alone (Figure 5I) did not affect the metabolic activity of cells. In all tests, cell viability oscillated around 100%, as can also be seen in Figure 6C for imatinib and nHAp/IM samples.

## 3. Materials and Methods

### 3.1. X-ray Powder Diffraction (XRPD)

The XRPD patterns obtained from nHAp and nHAp/IM were detected by using a PANalytical X’Pert Pro X-ray diffractometer (Malvern Panalytical Ltd., Royston, UK) equipped with Ni-filtered Cu Kα1 radiation (Kα1 = 1.54060 Å). All samples were measured under the same conditions, voltage: 40 kV, current: 30 mA, and a scan angle (2θ) in the range of 5° to 80° (step size = 0.0263°, time per step = 2.5 s). The experimental nHAp/IM diffractogram was compared with the pattern of nHAp standard from Inorganic Crystal Structure Database (ICSD–180315 [51]) with the pattern of unmodified imatinib supplied by Sigma Aldrich, as well as with the experimental diffractogram of IM.

The average crystallite size of nHAp was calculated based on the Rietveld refinement method [52] using the MAUD [53] program, version 2.93, based on the apatite hexagonal crystal structure with the better approximation and indexing using the Crystallographic Information File (CIF).

### 3.2. Scanning Electron Microscopy with Energy-Dispersive X-ray Spectroscopy (SEM-EDS)

The morphology and chemical composition of the samples were checked using a FE-SEM microscope FEI Nova NanoSEM 230 (FEI Company as a part of Thermo Fisher Scientific Inc., Hillsboro, OR, USA) equipped with an energy dispersive X-ray spectrometer (EDAX Genesis XM4). The samples were dispersed in alcohol, and then a drop was placed on the silicon stub. After drying using an infrared lamp, samples were put under the microscope. SEM-EDS measurements were carried out with an acceleration voltage of the 3.0 and 15.0 kV, respectively.

### 3.3. Absorption Spectroscopy

The absorption spectra were recorded on an Agilent Cary 5000 UV-Vis-NIR spectrophotometer (Agilent Technologies, Santa Clara, CA, USA) employing a spectral bandwidth of 0.1 nm in the ultraviolet-visible (UV-Vis) range. The spectra were recorded in the range of 230 to 450 nm (43,478–22,222 cm^−1^). The imatinib content in the nHAp/IM formulation was estimated from the calibration curve based on a series of known concentration solutions (0 to 50 μg/mL) of the drug at room temperature in 4% acetic acid (see Figure S2). The estimated concentration of IM (Analyte) amounted to 98 μg/mL, which was very close to the value derived from the IM stock solution (100 µg/mL).

The drug-loading capability (LC) and loading efficiency (LE) of nHAp/IM were evaluated by determining the total amount of IM in the suspension, and the IM loaded onto the nHAp surface using UV-Vis spectrophotometry. The LC and LE were calculated using the Equations (1) and (2), respectively.

(1) $$LC=\frac{W_{1}-W_{2}}{W_{3}}\times 100\;\%$$

(2) $$LE=\frac{W_{1}-W_{2}}{W_{1}}\times 100\;\%$$

where *W*_1_, *W*_2_, and *W*_3_ represent the total amount of IM, free drug in the supernatant, and nanoparticle weight, respectively.

#### Imatinib Release

The in vitro drug release study was carried out by the ultracentrifugation method in PBS buffer (pH 7.4). An appropriate amount of nHAp/IM was incubated in the releasing medium at 37 °C. The suspension was sampled at the predetermined time intervals: 0, 5, 10, 20, 30, and 45 min, as well as 1, 2, 6, and 24 h, and was then centrifuged (12,500 rpm, 5 min). The obtained supernatant was analyzed to determine the IM concentration with UV-Vis detection.

### 3.4. Fourier Transform Infrared Spectroscopy (FT-IR)

The FT-IR spectra of the imatinib, as well as the unmodified and modified nHAp, were performed using the KBr pellet method by using Nicolet iS50 from Thermo Fisher Scientific (Waltham, MA, USA) equipped with an Automated Beamsplitter exchange system (iS50 ABX containing a DLaTGS KBr detector and HeNe laser as the IR radiation source). The mid-IR spectra were collected in the 4000–400 cm^−1^ range, with spectral resolution equal to 2 cm^−1^.

### 3.5. Dynamic Light Scattering Particle Size Analysis (DLS) and Zeta (ζ) Potential

The hydrodynamic size distribution profile and surface charge quantification of obtained samples were evaluated by DLS and Zeta potential analyser Zetasizer Nano ZS apparatus from Malvern Instruments, operating under He-Ne 633 nm laser and equipped with the Dispersion Technology Software for data collection and data analysis. The suspensions of the nHAp and nHAp/IM were prepared by dilution with de-ionized water and appropriate cell-culture medium to eliminate errors connected with too high or too low amount of analyzed object and preserve the environment in which the cellular response to the material tested was studied. Each measurement was repeated three times to achieve reliable statistics. The hydrodynamic radius (r_h_) and zeta potential (ζ) of the particles in suspensions were determined using the Stokes–Einstein [54] and Henry’s [55] equations, respectively

(3) $$r_{h}=\frac{k_{B}T}{6\pi \eta D_{t}}$$

where *K_B_* is the Boltzmann’s constant, *T* is temperature, *D_t_* is the particle diffusion coefficient, and *η* is solvent viscosity.

(4) $$\zeta =\frac{U_{E}3\eta }{2\varepsilon f\left(K_{\alpha }\right)}$$

where *U_E_* is electrophoretic mobility, *ε* is the dielectric constant, *η* is solvent viscosity, and *f(K_a_*) is Henry’s function.

### 3.6. Biological Analysis

#### 3.6.1. Cell Cultures

The canine mastocytoma NI-1 cell line was kindly donated to us by Dr. med. vet. Emir Hadzijusufovic from the Clinic for Internal Medicine and Infectious Diseases, University of Veterinary Medicine Vienna, Vienna, Austria. NI-1, non-adherent cells, were grown in suspension in RPMI-1640 medium (Sigma-Aldich, St. Louis, MO, USA), supplemented with 10% fetal bovine serum (FBS). Murine fibroblasts L929 (American Type Culture Collection, ATCC) control cells were cultured in Minimum Essential Medium Eagle (MEM, Sigma-Aldich, St. Louis, MO, USA) containing 10% FBS (Sigma-Aldich, St. Louis, MO, USA). The canine osteosarcoma D17 control cell line (European Collection of Authenticated Cell Cultures, ECACC) was cultured in modified Eagle’s medium (MEM) supplemented with F12 Ham’s nutrient an 10% FBS. All the cells were cultured in standard conditions (humidified incubator, 37 °C, 5% CO_2_).

#### 3.6.2. Cytotoxicity Assay

The cytotoxic effect of imatinib alone, as well as unmodified and modified nHAp was measured with an Alamar blue assay (TOX-8 in vitro Toxicology Assay Kit, Sigma-Aldich, St. Louis, MO, USA), which is a technique that measures colorimetrically the amount of resazurin reduced by metabolically active cells. The procedure was carried out according to the protocol provided by the manufacturer. Briefly, NI-1 and L929 cells were seeded on 96-well plates at a density of 5000 cells per well in 100 µL of dedicated medium and cultured overnight in standard conditions. Then, cells were incubated with test compounds (imatinib, modified and unmodified nHAp) at concentrations from 1 × 10^−11^ to 2.29 × 10^−5^ M (imatinib) and 1 × 10^−10^ to 1 × 10^−10^ g/mL (nHAp) for 48 h under standard conditions. Afterward, media from cell cultures were replaced with 10% *v*/*v* TOX8 dye solution in full medium. Cells were incubated in standard conditions for 2 h, and the spectrophotometric measurement was performed using a microplate reader (Epoch BioTek^®^, Winooski, VT, USA). Spectrophotometric reading was evaluated at 600/690 nm wavelengths. As blank, full medium supplemented with 10% *v*/*v* dye solution was used. Statistical analysis was determined using GraphPad Prism 5.01 (San Diego, CA, USA). Statistical significance was analyzed using an unpaired *t*-test and *p* values < 0.05 were considered statistically significant. The results shown in figures represent mean values ± standard deviation (SD). *p* values less than 0.05 (*p* < 0.05), *p* < 0.01 and *p* < 0.001 were summarized with one (*), two (**) or three asterisks (***), respectively.

## 4. Conclusions

The aim of this study was to evaluate the possibility of combining nanohydroxyapatite with an anticancer drug with known efficacy as well as the characteristics of such preparations in terms of both physicochemical properties and therapeutic potency. As a result of conducted experiments, a combination of nanocrystalline material and an amorphous anticancer drug were obtained.

The results suggested that this combination interacted through surface adsorption. It has been shown that imatinib did not change the structure of the nanomaterial, but it affected such properties as particle size distribution in the medium and surface charge-important parameters for cell-nanoparticle interaction. Whereas, the drug conversion from crystalline to amorphous form during the experimental conditions contributed to its better solubility, and thus bioavailability.

The study confirmed the biocompatibility of nHAp alone and demonstrated that its combination slightly improved the effectiveness of the attached drug. Considering these results, undoubtedly, the combination of drugs with the proven efficacy with nHAp as a nanoscale drug carrier might be an idea worth developing and should be further researched.

## Figures and Tables

**Table 1:** Unit cell parameters (a, c), cell volume (V), crystallite size, and refine factor (R_wp_) for the Ca_10_(PO_4_)_6_(OH)_2_ (nHAp) prepared by co-precipitation method and annealed at 500 °C.

Sample	a (Å)	c (Å)	V (Å^3^)	Size (nm)	R_wp_ (%)
single crystal [40]	9.424(4)	6.879(4)	529.09(44)	–	–
nHAp	9.430(4)	6.891(4)	530.76(02)	45.5(5)	3.2

**Table 2:** Zeta potentials of nanohydroxyapatite (nHAp), Imatinib mesylate (IM), and nHAp modified by IM in de-ionized water and culture medium.

Sample	Zeta Potential (*mV*) De-Ionized Water	Zeta Potential (*mV*) Cell Culture Medium
nHAp	−18.8 ± 0.2	−10.4 ± 0.3
IM	−15.9 ± 0.7	−8.9 ± 0.5
nHAp/IM	−14.0 ± 0.4	−10.4 ± 0.1

## Supplementary Materials

The following are available online, Figure S1: Time dependent of imatinib release from the drug loaded nHAp/IM.; Figure S2: Evaluation of imatinib (Analyte) concentration in the nHAp/IM (4-fold dilution in relation to the stock solution).
