# Supplementary material for: Nanohydroxyapatite-Mediated Imatinib Delivery for Specific Anticancer Applications
Source: Molecules. 2020 Oct 9;25(20):4602. doi: 10.3390/molecules25204602 (PMC7587182; doi:10.3390/molecules25204602)
Supplement: Supplementary file 1 [file molecules-25-04602-s001.pdf]

molecules

Supplementary information

Nanohydroxyapatite-mediated imatinib delivery for specific anticancer applications

Paulina Sobierajska [1,*], Anna Serwotka-Suszczak [2], Damian Szymanski [1], Krzysztof Marycz [2,3,4], and Rafal J. Wiglusz [1,3,*]

1 Institute of Low Temperature and Structure Research, Polish Academy of Sciences, Okolna 2, 50-422 Wroclaw, Poland; d.szymanski@intibs.pl
2 Department of Experimental Biology, Wroclaw University of Environmental and Life Sciences, Norwida 27B street, A7 building, 50-375 Wroclaw, Poland; anna.serwotka-suszczak@upwr.edu.pl (A.S.-S.); krzysztofmarycz@interia.pl (K.M.)
3 International Institute of Translational Medicine, Malin, Jesionowa 11, 55-114 Wisznia Mała, Poland
4 Faculty of Veterinary Medicine, Equine Clinic-Equine Surgery, Justus-Liebig-University, 35392, Giessen, Germany
* Correspondence: p.sobierajska@intibs.pl (P.S.); r.wiglusz@intibs.pl (R.J.W.); Tel.: +48-071-3954-274 (P.S.); +48-071-3954-159 (R.J.W.)

Academic Editor: Derek J. McPhee
Received: 9 September 2020; Accepted: 7 October 2020; Published: 9 October 2020

2. *Results and discussion*

2.1. *Characterisation of nHAp, Imatinib and nHAp/Imatinib*

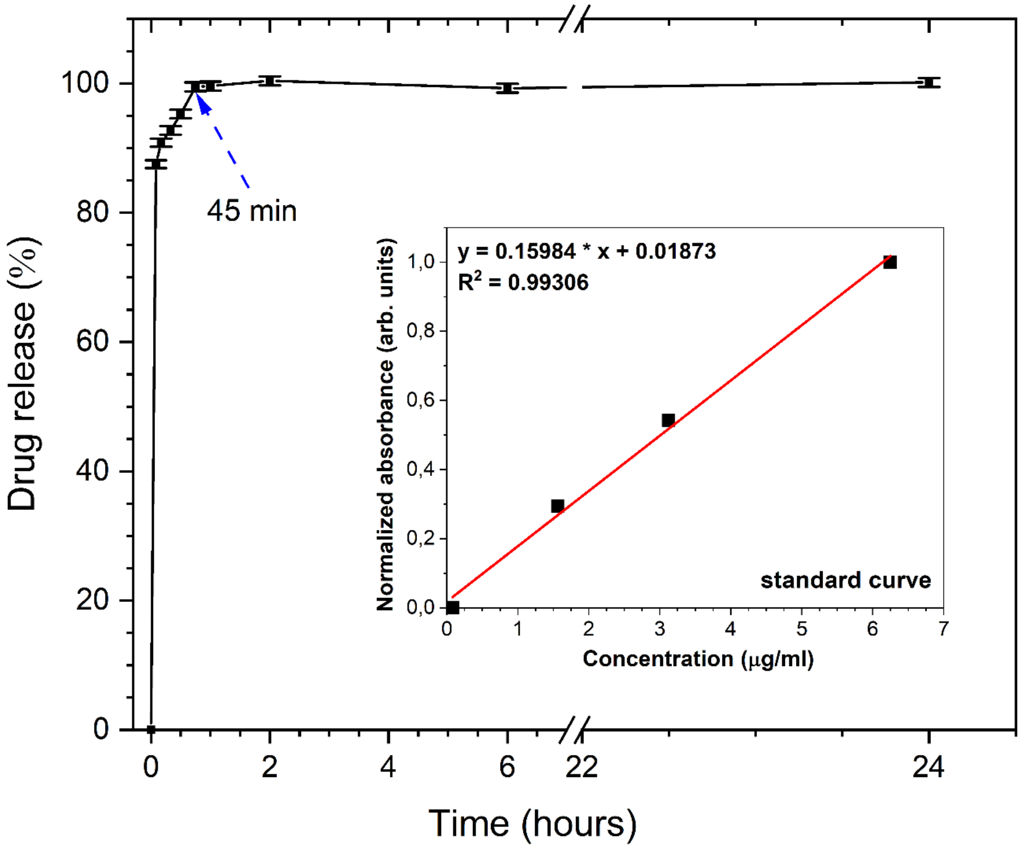

Figure S1. Time dependent of imatinib release from the drug loaded nHAp/IM.

molecules

MDPI

3. *Materials and Methods*

3.3. Absorption spectroscopy

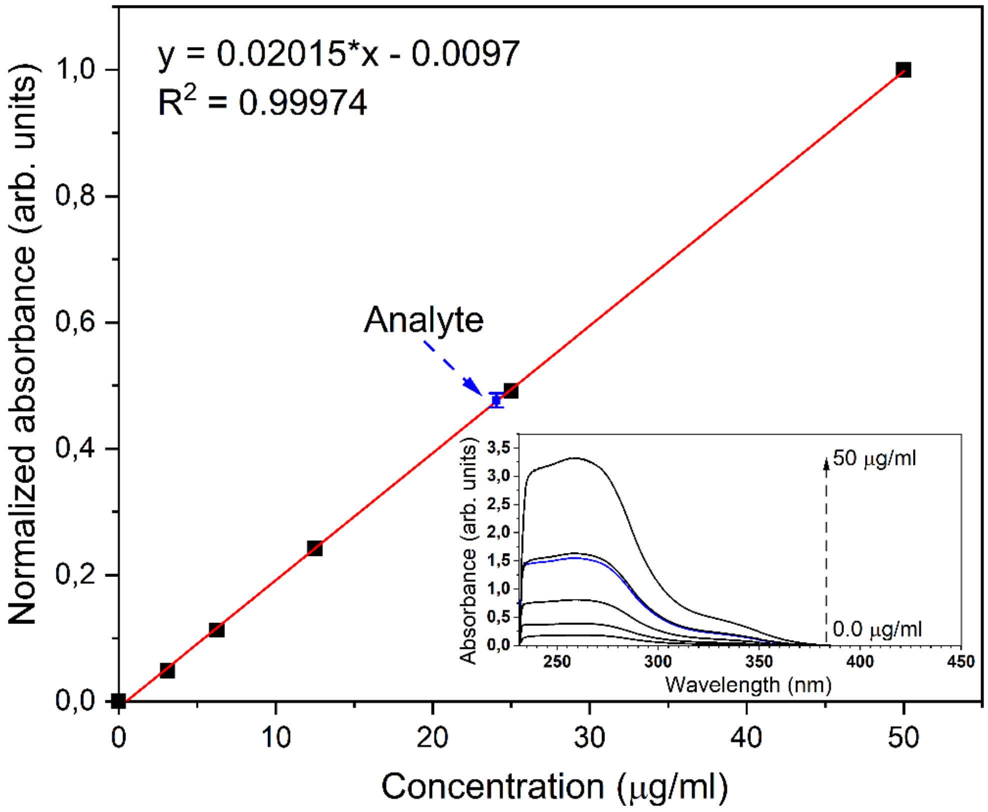

Figure S2. Evaluation of imatinib (Analyte) concentration in the nHAp/IM (4-fold dilution in relation to the stock solution).
